# Structure of precursor microRNA’s terminal loop regulates human Dicer’s dicing activity by switching DExH/D domain

**DOI:** 10.1007/s13238-014-0124-2

**Published:** 2014-12-31

**Authors:** Zhongmin Liu, Jia Wang, Gang Li, Hong-Wei Wang

**Affiliations:** 1 Department of Biochemistry and Molecular Biology, School of Basic Medical Sciences, Peking University Health Science Center, Beijing, 100191 China; 2 Tsinghua-Peking Joint Center for Life Sciences, School of Life Sciences, Tsinghua University, Beijing, 100084 China

**Keywords:** human Dicer, DExH/D (ATPase-helicase) domain, pre-miRNA-151, A-to-I editing, single particle electron microscopy

## Abstract

Almost all pre-miRNAs in eukaryotic cytoplasm are recognized and processed into double-stranded microRNAs by the endonuclease Dicer protein comprising of multiple domains. As a key player in the small RNA induced gene silencing pathway, the major domains of Dicer are conserved among different species with the exception of the N-terminal components. Human Dicer’s N-terminal domain has been shown to play an auto-inhibitory function of the protein’s dicing activity. Such an auto-inhibition can be released when the human Dicer protein dimerizes with its partner protein, such as TRBP, PACT through the N-terminal DExH/D (ATPase-helicase) domain. The typical feature of a pre-miRNA contains a terminal loop and a stem duplex, which bind to human Dicer’s DExH/D (ATPase-helicase) domain and PAZ domain respectively during the dicing reaction. Here, we show that pre-miRNA’s terminal loop can regulate human Dicer’s enzymatic activity by interacting with the DExH/D (ATPase-helicase) domain. We found that various editing products of pre-miR-151 by the ADAR1P110 protein, an A-to-I editing enzyme that modifies pre-miRNAs sequence, have different terminal loop structures and different activity regulatory effects on human Dicer. Single particle electron microscopy reconstruction revealed that pre-miRNAs with different terminal loop structures induce human Dicer’s DExH/D (ATPase-helicase) domain into different conformational states, in correlation with their activity regulatory effects.

**Electronic supplementary material:**

The online version of this article (doi:10.1007/s13238-014-0124-2) contains supplementary material, which is available to authorized users.

## Introduction

MicroRNAs are emerging as an important regulator of almost all biological processes ranging from cell differentiation to senescence (He and Hannon, 2004; Schraml and Grillari, 2012). Thousands of precursor microRNAs are precisely processed into microRNAs with a typical length of 20–25 nt by RNase III enzyme Dicer (Griffiths-Jones et al., 2008;  Schraml and Grillari, 2012). The multi-domain Dicer is conserved in all eukaryotic species with the RNase domain, PAZ domain and dsRBD domain but differ in their N-terminal compositions (Carthew and Sontheimer, 2009). The human Dicer (hDicer) with 220 kDa contains an N-terminal DExH/D (ATPase-helicase) domain, a DUF283 domain, a PAZ domain, two RNase-III domain and a double-stranded RNA-binding domain (dsRBD) (Carthew and Sontheimer, 2009). The two RNase-III domains form an intra-molecular dimer to function as an endonuclease processing center for double-stranded RNA substrates (Zhang et al., 2004).

The typical secondary structure of a pre-miRNA contains a terminal loop and a stem with the 5′ phosphate and 2-nucleotide (nt) 3′ overhang. In a pre-miRNA maturation process, Dicer’s PAZ domain recognizes and binds with the 5′ phosphate and 2-nucleotide (nt) 3′ overhang of a pre-miRNA (Park et al., 2011), and the N-terminal DExH/D (ATPase-helicase) domain interacts with the terminal loop of pre-miRNA (Tsutsumi et al., 2011) to align the pre-miRNA to the RNase III site for precise cleavage. The terminal loop sequence and structure of pre-miRNA could directly impact on the binding affinity of RNA to Dicer protein and even on the cleavage of Dicer (Zhang and Zeng, 2010; Castilla-Llorente et al., 2013). Furthermore, the terminal loop also plays a critical role for accuracy of Dicer processing (Gu et al., 2012).

Recently, non-canonical pre-miRNAs with 5′-cap termini (Xie et al., 2013), 5′ overhanging termini (Ando et al., 2011), and uridylated termini (Heo et al., 2008; Heo et al., 2012) were reported to be diced into mature miRNAs. In order to accurately processing thousands of pre-miRNAs with different features into mature miRNAs, the only single isoform of Dicer in human cells (Griffiths-Jones et al., 2008) needs precise activity regulation upon different miRNA precursors in various cellular environment. The N-terminal DExH/D (ATPase-helicase) domain has been shown to auto-inhibit the dicing activity of hDicer (Ma et al., 2008). Human Dicer’s regulatory proteins such as TRBP (Chendrimada et al., 2005), PACT (Yoontae Lee et al., 2006), and ADAR1 (Ota et al., 2013) interact with hDicer via its N-terminal DExH/D (ATPase-helicase) domain, and increasing dicing activity has been observed within these heterodimers. The regulatory mechanism of the DExH/D (ATPase-helicase) domain on hDicer’s activity, however, remains elusive.

Post-transcriptional RNA editing introduces the nucleotide changes in specific RNA sequence in tRNA, rRNA and miRNA and also plays an important role in gene expression (Axel Brennicke et al., 1999; A. A. H. Su and L.Randau, 2011). ADAR family of enzymes mediate adenosine to inosine changes in double-stranded RNAs, contributing the major form of RNA editing events in human cells (Nishikura, 2010). ADAR-mediated RNA editing of the double-stranded portion of primary and precursor microRNAs were shown to result in the redirection of the silencing target of microRNA (Blow et al., 2006; Das and Carmichael, 2007; Kawahara et al., 2007b). How and why the sequence modification of miRNA precursors affect their silencing property are yet to be fully understood.

One of the ADAR family protein, ADAR1P110, was shown to induce the RNA editing of pre-miR-151 to generate three different products, pre-miR-151A1I, pre-miR-151A3I and pre-miR-151A13I by both *in vivo* and *in vitro* assays (Kawahara et al., 2007a). These products were reported to bind with hDicer-TRBP complex but cannot be further diced into mature miR-151. Until now, there is little explanation for this observation. In this work, we discovered that edited and unedited pre-miR-151 can directly bind with hDicer. *In vitro* dicing assay shows that while pre-miR-151A1I and pre-miR-151A13I were processed with greatly reduced dicing rate compared to pre-miR-151, the dicing of pre-miR-151A3I was not much affected. Single particle electron microscopy reconstruction showed that hDicer-pre-miR-151 and hDicer-pre-miR-151A3I complex has a similar 3D structure with the DExH/D (ATPase-helicase) domain in an open state. In comparison, the DExH/D (ATPase-helicase) domain in the hDicer-pre-miR-151A1I and hDicer-pre-miR-151A13I complexes appeared in a close state. The structural variation in good correlation with our dicing activity assay results indicated that the pre-miRNA’s terminal loop structure has a strong regulatory effect of hDicer’s activity via induced conformational change of the protein’s DExH/D (ATPase-helicase) domain.

## Results

### Electron microscopy of human Dicer with a well-defined N-terminal DExH/D domain

**Figure 1:**
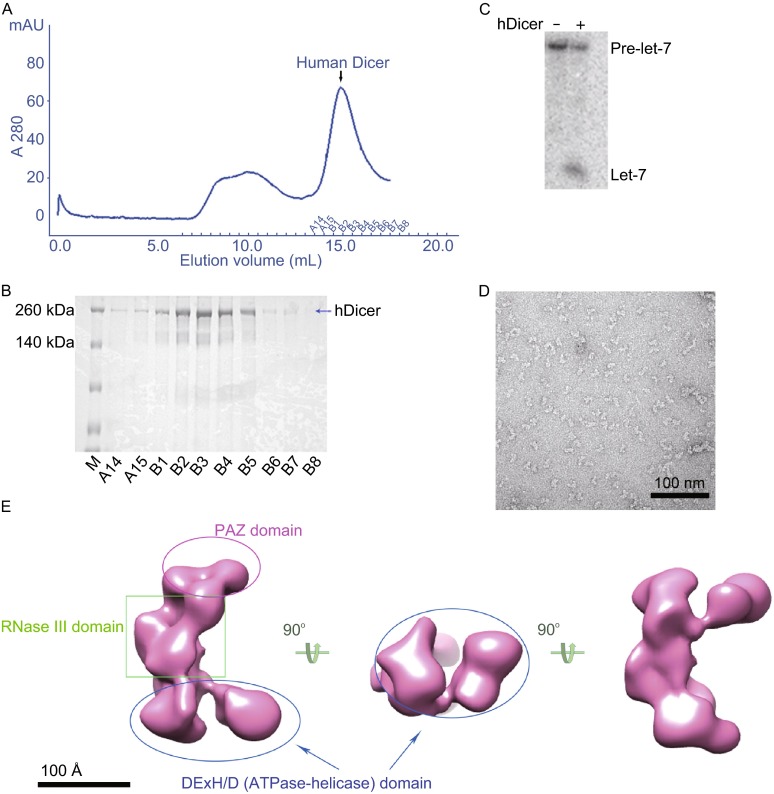
**Purification and 3D reconstruction of human Dicer protein**. (A and B) hDicer protein fractions eluted from size exclusion column (Superose 6 10/300 GL) and 8% SDS-PAGE gel of the fractions stained by Coomassie brilliant blue. (C) hDicer’s dicing assay on 60 nmol/L radioactive labeled pre-let-7. (D) Raw images of apo-hDicer was recorded with CCD at a nominal magnification of 49,000×, defocus value of −1 to −3 µm. (E) 3D reconstruction of hDicer protein. The location of domains are labeled in the model

We expressed and purified hDicer using the insect cell system to high purity (Fig. 1A and 1B). The purified hDicer was verified to have endonuclease activity on its classical pre-miRNA substrate, pre-let-7 (Fig. 1C). We examined the purified hDicer using negative staining transmission electronic microscopy (TEM) and found that the protein were mono-dispersed and homogenous in shape and dimension (Fig. 1D). We thus performed single particle reconstruction of hDicer from 50,403 particle images to verify the protein’s structural features with previously reconstructed models (Wang et al., 2009; Lau et al., 2012; Taylor et al., 2013). This practice also set a consistent protocol for the EM study of the different RNA-hDicer complexes as discussed below. Different from the previous hDicer reconstructions, we used the most recent version of RELION image processing package (Scheres, 2012) to perform maximum-likelihood 3D classification of the particle images in order to overcome the conformational heterogeneity and negative stain artifact problem of hDicer as revealed before (Taylor et al., 2013). We found the latter to be the major issue in our structural determination. After classification of all the particles into four classes, we found that all the four classes appeared to share similar L-shape as the previous reported 3D reconstructions (Lau et al., 2012; Taylor et al., 2013), but the third class had the best defined features and the highest resolution and was composed of the majority of particles (Figs. 1E and S1). The structure revealed a clear shape of the N-terminal DExH/D domain that forms a V-shaped arrangement with hDicer’s RNase domains from the bottom view of the molecule. We found that the change of this structural arrangement of DExH/D (ATPase-Helicase) domain reflects the interaction of different pre-miRNA substrates with hDicer as described below.

### RNA editing causes different pre-miRNA terminal loop structures

**Figure 2:**
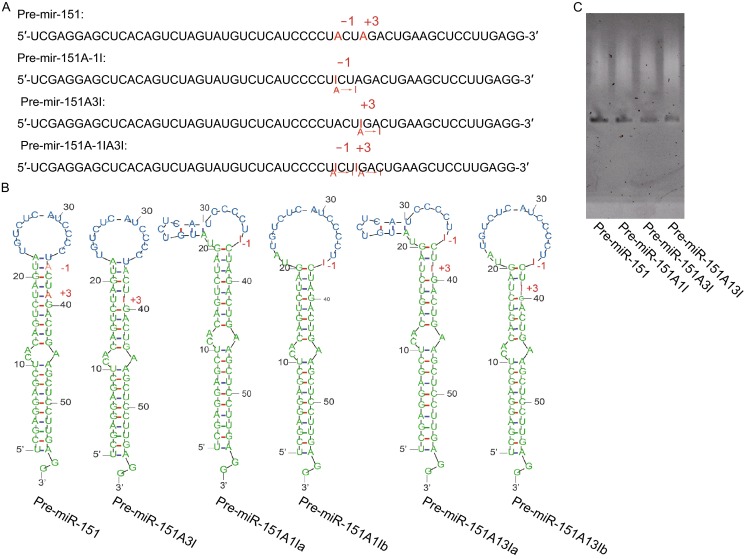
**The RNA editing sites on pre-miR-151 and the secondary structure of edited and unedited pre-miR-151**. (A) Pre-miR-151 and its A-to-I edited partners. Green boxes represent the sequences in the diced product. −1 Stands for the first nucleotide outside the dicing site; +3 represents the third nucleotide inside the dicing site from 5′ end. −1 And +3 labelled adenosine can be converted into inosine by ADAR1P110. (B) Secondary structural prediction of the unedited and edited pre-miR-151 RNAs using Mfold web server. Note the two possible structures of terminal loop of pre-miR-151A1I and pre-miR-151A13I. (C) Native gel electrophoresis of synthesized pre-miR-151 and its edited partners

ADAR enzymes deaminate the adenosine into inosine (A-to-I) within dsRNAs (Nishikura, 2010). It is known that ADAR deaminase can modulate the processing and expression of microRNA and redirect the silencing targets (Blow et al., 2006; Kawahara et al., 2007b). Human ADAR1P110 edits pre-miR-151 to generate three major products based on the A-to-I editing positions relative to the dicing site on the RNA stem, namely, pre-miR-151A1I at −1 site, pre-miR-151A3I at +3 site, and pre-miR-151A13I at −1 and +3 sites, respectively (Fig. 2A) (Kawahara et al., 2007a). We exploited secondary structure prediction of the four types of RNA molecules including the unedited and the three edited pre-miR-151 products using Mfold RNA structural folding server (Zuker, 2003). The structural prediction showed that A-to-I changes at position +3 have little effect on the secondary structure of pre-miR-151. In contrast, the A-to-I editing at position −1 clearly destabilizes the stem end close to the loop therefore generates different terminal loop structures from that of the unedited pre-miR-151. As a result, the edited product pre-miR-151A3I adopts a similar terminal loop structure with pre-miR-151, whereas pre-miR-151A1I and pre-miR-151A13I may have either a big loop or a hammerhead-shaped terminal loop (Fig. 2B). In order to study these RNAs’ transactions with human Dicer, we synthesized the pre-miR-151 and its A-to-I editing products which all appeared as single folding species on native gel electrophoresis (Fig. 2C).

### Human Dicer has different dicing activity on edited pre-miR-151

**Figure 3:**
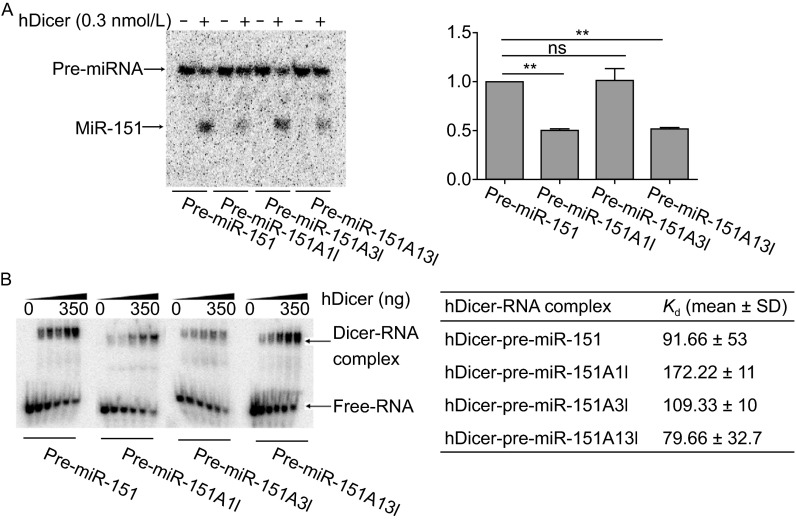
***In vitro*** **cleavage of hDicer and RNA binding analysis**. (A) Dicing assay of pre-miR-151 and its A-to-I edited partners by human Dicer. The ratio of diced product microRNA-151 against precursor substrate pre-miR-151 was normalized to ʺ1ʺ. Data present mean ± SD (*n* = 3). (**) were highly significant when compared with the substrate pre-miR-151, in both cases *P *< 0.002. (B) EMSA assay of pre-miR-151 and its A-to-I edited partners binding to human Dicer. Varying amounts of purified hDicer (0, 7, 14, 35, 70 and 350 ng) binding to edited and unedited pre-miR-151 were examined by electrophoresis mobility shift assay using a native 6% polyacrylamide gel. Data present mean ± SD (*n* = 3)

In order to reveal the effect of A-to-I editing on pre-miRNA’s processing activity by hDicer, we performed *in vitro* dicing activity assay of hDicer over pre-miR-151 and its related A-to-I editing products. Using 5′ radioactive ^32^P labeled pre-miR-151, pre-miR-151A1I, pre-miR-151A3I and pre-miR-151A13I, we found that the RNA editing on position +3 adenosine had little effect on hDicer’s dicing activity of miR-151 compared to that of unedited substrate, but editing on position −1 or both −1 and +3 adenosine greatly reduced the cleavage activity of hDicer (Fig. 3A). It is possible that the different dicing activity may be due to the various binding affinity of hDicer with the RNA substrates. We therefore performed electrophoresis mobility shift assay (EMSA) to examine the interaction of hDicer with different RNA substrates. Our results suggested that, within the error range of the assays, the edited pre-miR-151A1I, pre-miR-151A3I and pre-miR-151A13I have approximately the same *K*d value as the unedited pre-miR-151 for binding to hDicer (Fig. 3B). This also indicates that the different dicing activities of various RNA species by hDicer are likely due to the effect on hDicer directly by the secondary structural difference of terminal loops of the RNAs.

### RNA editing results in conformational change of hDicer

**Figure 4:**
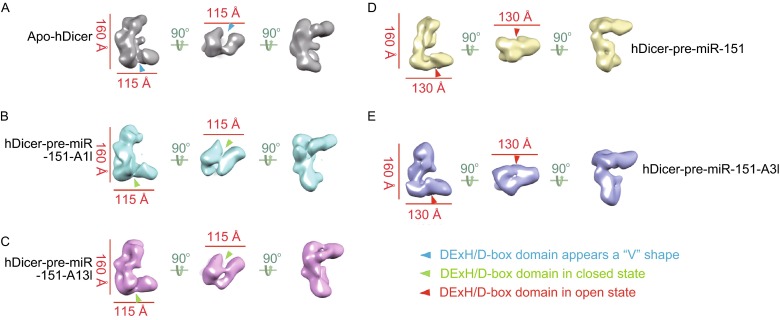
**3D reconstruction of hDicer-RNA complexes**. (A) 3D structures of human apo-Dicer appear as an L shape with the DExH/D (ATPase-helicase) domain like a “V” shape, and the distance between two branches of DExH/D (ATPase-helicase) domain is about 115 Å. (B and C) hDicer-pre-miR-151A1I complex and hDicer-pre-miR-151A13I complex appear as an L shape with DExH/D (ATPase-helicase) domain in a close state. The distance between two branches of DExH/D (ATPase-helicase) domain is less than 115 Å. (D and E) DExH/D (ATPase-helicase) domain in hDicer-pre-miR-151 complex and hDicer-pre-miR-151A1I complex appears an open state. The distance between two branches of DExH/D (ATPase-helicase) domain is about 130 Å

We have shown previously that pre-let7 and dsRNA precursors induce hDicer into distinct conformations and thus cause hDicer’s different activity on these substrates (Taylor et al., 2013). In order to investigate the mechanism of RNA editing’s influence on hDicer’s activity, we performed single particle analysis of hDicer incubated with edited and unedited RNA substrates at 4°C and in the presence of 2 mmol/L EDTA, a condition under which hDicer protein has no endonuclease activity (Zhang et al., 2002) but can still form stable complex with RNA. Using similar single particle analysis approaches as for hDicer only, we obtained the 3D reconstructions of hDicer in complex with pre-miR-151, pre-miR-151A1I, pre-miR-151A3I and pre-miR-151A13I, respectively, using the same analysis procedure as for apo-hDicer, i.e. classifying the particles into four classes and choosing the best model for further analysis (Fig. 4). Overall shapes of all the structures are similar as that of hDicer only but close examination revealed interesting conformational difference, especially in the N-terminal DExH/D (ATPase-helicase) domain.

Compared with the hDicer only structure, pre-miR-151-bound-hDicer adopts a dramatic conformational change of the N-terminal domain which appears to rotate into a perpendicular arrangement with the RNase domain (Fig. 4D). Such a conformational change causes the elongation of hDicer’s base-branch from ~115 Å in the apo-hDicer to ~130 Å in the RNA-bound hDicer. We termed this conformation as the open state of hDicer. A feature worth noting of the open-state is a density filling the space between the DExH/D and RNase domain (pointed by the red arrowheads in Fig. 4D and 4E). Another conformational variant happens in the complex of hDicer and pre-miR-151A1I, in which the N-terminal domain rotates into a more parallel arrangement with the RNase domain and makes the space between the DExH/D and RNase domain very narrow (Fig. 4B). We termed this conformation the close state of hDicer.

Interestingly, the RNA substrate induced conformational changes are correlated with the specific structural feature of the RNA terminal loops. It was obvious that the pre-miR-151 and pre-miR-151A3I with the same predicted terminal loop structure both induced hDicer into the open state (Fig. 4D and 4E). In contrast, the pre-miR-151A1I and pre-miR-151-A13I with a different predicted terminal loop structure from that of pre-miR-151 both induced hDicer into the close state (Fig. 4B and 4C). Such a conformational change variation also correlated to the dicing activities of these substrates by hDicer as shown earlier. Therefore, the terminal loop structural change caused by A-to-I edition of pre-miR-151 induces specific conformational change of hDicer that represents the enzyme’s different activity states.

## Disscussion

ADAR1P110 is a constitutive expressed isoform of ADAR1 protein which are proved to be a homo-dimer when functions as an RNA editing enzyme (Nishikura, 2010). ADAR1 mediated A-to-I deamination reaction on dsRNA is the major RNA editing form in human cells (Blow et al., 2006; Kawahara et al., 2007b; Nishikura, 2010). Pri-miRNA and pre-miRNA share a feature structure of dsRNA which is susceptible to be substrates of ADAR1 family (Nishikura, 2010). Many pri-miRNA and pre-miRNA are predicted to be targets of ADAR1 proteins and some pri-miRNA and pre-miRNA, for example pre-miR-142 (Yang et al., 2006), pri-miR-151 (Kawahara et al., 2007a) and pri-miR-376 (Kawahara et al., 2007b), have been proved to be targets of ADAR1 by biochemical assays. The adenosine converted into inosine happening near the terminal loop of pre-miR-151 could make terminal structure rearranged following the nucleotide changed in terminal loop (Fig. 2B). Our structural prediction data confirmed the structural changes of terminal loop within edited pre-miR-151. The nucleotide sequence and structure of the terminal loop have been shown to play a critical role in the cleavage of pre-microRNA (Zhang and Zeng, 2010; Gu et al., 2012; Castilla-Llorente et al., 2013). Agreeing with the terminal loop structure’s importance, our *in vitro* cleavage assay showed that the pre-miR-151A1I and pre-miR-151A13I with changed terminal structure reduced the endonuclease activity of hDicer (Fig. 3B), while the A-to-I editing at +3 site only with little terminal loop structural change does not affect hDicer’s activity.

**Figure 5:**
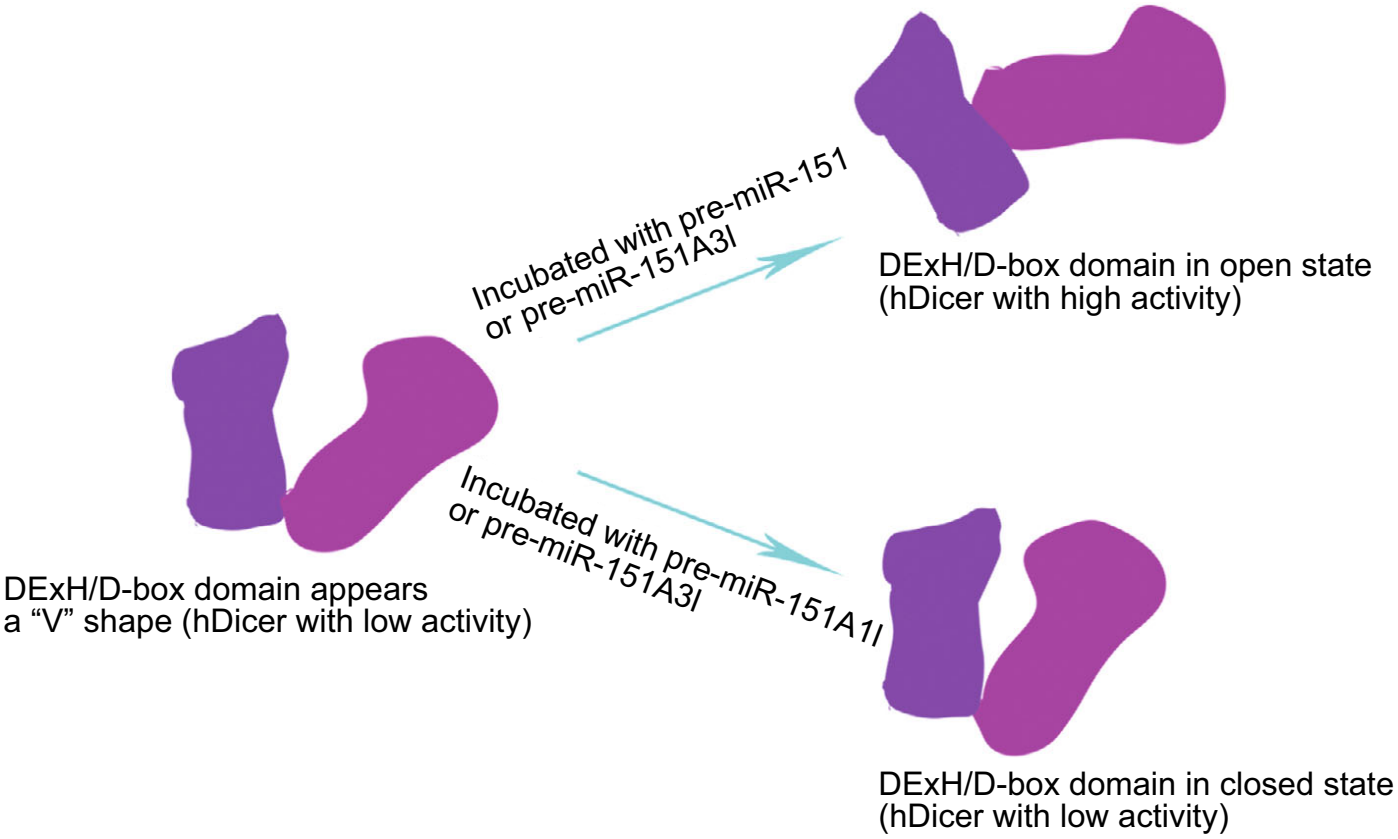
**Model of DExH/D (ATPase-helicase) domain regulating the dicing activity of human Dicer protein**. From the bottom view of human Dicer as in Fig. 4, the relative position between the branch (pink) relative to the main body (purple) of human Dicer switches depending on the RNA substrate’s different loop structures

In human Dicer, the N-terminal DExH/D (ATPase-helicase) domain plays an important auto-inhibition regulatory function on the enzyme’s dicing activity (Ma et al., 2008). Our previous work has shown that pre-miRNA and dsRNA substrates interacting differently with the N-terminal domain induce hDicer into dramatic different conformations either in an inhibitory state or an active state (Taylor et al., 2013). We also showed how hDicer’s interaction cofactor proteins such as TRBP and PACT may enhance the protein’s activity by changing its N-terminal domain conformation. In the current work, for the first time, we dissected the regulatory mechanism of hDicer’s activity by structural elements of the pre-miRNA. Our results combining the structural prediction, *in vitro* dicing activity assays, and single particle EM reconstruction revealed a strong correlation of pre-miRNA’s terminal loop structure with its effect on hDicer’s conformation and activity (Fig. 5). Although the resolution of our 3D reconstruction at current stage is not high enough for us to see the RNA bound to hDicer, the dramatic conformational change induced by pre-miR-151 and its edited partners with different terminal loop structures may well reflect the terminal loop’s effect on hDicer’s N-terminal domain via direct interaction. The pre-miRNA terminal loop on one end induces hDicer’s N-terminal domain structural change, on the other end, helps pre-miRNA’s stem region align against hDicer’s RNase domain. Structural changes in terminal loop thus probably have an allosteric effect of the RNA substrate’s precise recognition by hDicer’s RNase site. A seemingly small change such as the A-to-I mutation at −1 site of pre-miR-151 could cause a dramatic structural variation of the stem terminal loop, therefore resulting the drop of hDicer’s processing activity. In contrast, the A-to-I mutation at +3 site of pre-miR-151, which does not affect the terminal loop’s structure, showed almost no effect on hDicer’s processing activity. This further underlines the importance of pre-miRNA’s terminal loop structure in regulating hDicer activity. Our discovery here that the structural changes of terminal loop within pre-miRNA correlating the switches of DExH/D (ATPase-helicase) domain of hDicer regulate hDicer’s activity provides insight into our understanding of how human Dicer processing pre-microRNA and opens new questions about miRNA terminal loops role in gene silencing regulation.

## Materials and methods

### Protein expression and purification

Human dicer was expressed and purified as described (MacRae et al., 2008). Briefly, Bac-to-Bac baculovirus expression system (Life technologies) was used to express the N-terminally His_6_-tagged human Dicer proteins. SF-21 cells were infected for 60 h with hDicer recombinant baculovirus before collecting them for sonication. Supernatant was applied to Ni^2+^-NTA resin after centrifugation at 3500 rpm for 1 h. Eluted hDicer protein was dialyzed with TEV enzyme against dialysis buffer overnight. The cleaved product was then applied to a 5 mL His Trap HP column, from which the flow-through was collected as the fraction containing His6-tag deleted hDcier. The protein was then concentrated to 1 mL and applied to a Sepharose 6 10/300 column for a final gel filtration step. The eluted protein solution in aliquots was snap-frozen in liquid nitrogen for storage at −80 centigrade.

### Synthetic RNA substrates

All of our RNA substrates were synthesized by TAKARA BIOTECHNOLOGY. Human pre-let-7: 5′-UGAGGUAGUAGGUUGUAUAGUUUUAGGGUCACACCCACCACUGGGAGAUAACUAUACAAUCUACUGUCUUACC-3′, human pre-miR-151: 5′-CGAGGAGCUCACAGUCUAGUAUGUCUCAUCCCCUACUAGACUGAAGCUCCUUGAGG-3′, human pre-miR-151A1I: 5′-UCGAGGAGCUCACAGUCUAGUAUGUCUCAUCCCCUICUAGACUGAAGCUCCUUGAGG-3′, human pre-miR-151A3I: 5′-UCGAGGAGCUCACAGUCUAGUAUGUCUCAUCCCCUACUIGACUGAAGCUCCUUGAGG-3′, human pre-miR-151A13I: 5′-CGAGGAGCUCACAGUCUAGUAUGUCUCAUCCCCUICUIGACUGAAGCUCCUUGAGG-3′. The RNA substrates were annealed by fast-cooling to fold them into the right structure. They were verified for purity and the correct folding by Gelsafe staining (Yuanpinghao Bio) after native gel electrophoresis and 5′-end-labeled with γ-^32^P-ATP (PerkinElmer) using T4 polynucleotide kinase (from TAKARA BIOTECHNOLOGY) before all related assays described in this work.

### *In vitro* cleavage of RNA substrates

The *in vitro* cleavage assay of different RNA substrates by hDicer were performed at 37°C for 1 h in 15 µL of reaction volume containing 60 nmol/L Dicer, 60 nmol/L γ-^32^p-labeled RNA substrates, 30 mmol/L Tris-HCl (pH 6.8), 50 mmol/L NaCl, 3 mmol/L MgCl_2_, 0.1% Triton X-100, 15% glycerol, 1 mmol/L DTT. Reactions were stopped with RNA loading buffer containing 8 mol/L Urea, 1× TBE, 0.05% Bromphenol blue and 0.05% Xylene cyanol, boiled for 4 min, then chilled on ice. RNA products were separated on an 18% polyacrylamide, 8 mol/L urea denaturing gel, visualized on a phosphor screen (Amersham Biosciences) with a Typhoon Trio Imager (Amersham Biosciences).

### Electrophoretic mobility shift assay (EMSA)

For EMSA, RNA substrates were incubated with hDicer on ice for 40 min in a 20 µL volume containing 30 mmol/L Tris-HCl (Ph 6.8), 25 mmol/L NaCl, 2 mmol/L MgCl_2,_ 1 mmol/L DTT and 2 mmol/L EDTA. Samples were resolved through 6% native acrylamide gels in 0.5× Tris-glycine buffer under an electric field of 15 V/cm for 70 min visualized on a phosphor screen (Amersham Biosciences) with a Typhoon Trio Imager (Amersham Biosciences).

### Negative staining electron microscopy

Human Dicer-RNA complexes were assembled by pre-incubating 150 nmol/L hDicer with a 500% excess of unedited and edited pre-miR-151 in a specific volume containing 20 mmol/L Tris-HCl, pH 6.8, 25 mmol/L NaCl, 2 mmol/L MgCl_2,_ 1 mmol/L DTT and 2 mmol/L EDTA and 5% glycerol. All the samples were negatively stained on holey carbon grids covered by a thin layer of continuous carbon over holes with 2% (*w*/*v*) uranyl acetate solution. All micrographs were collected on a Tecnai F20 Twin transmission electron microscope (FEI) running at 200 kV or an Tecnai-12 Biotwin electron microscope (FEI) operated at 120 kV, using a nominal magnification of 50,000× or 49,000×, respectively. The micrographs were collected on Ultrascan 4000 CCD camera (Gatan Inc.) at specimen-level pixel sizes of 0.223 nm or 0.29 nm, respectively, using a dose of ~30 e^−^ Å^−2^ and a nominal defocus range of −1 to −3 μm.

### Image processing and model reconstructions

For 3D classification of apo-hDicer and hDicer-RNA complexes, about 70,000 particles of each sample were picked together from each set of micrographs. EMAN2 (Tang et al., 2007) was used to do the particle picking. Two-dimensional classification and alignment of particle images were performed using IMAGIC-4D (van Heel et al., 1996). Three dimension classification and refinement was performed with Relion1.2 (Scheres, 2012). The starting 3D model was generated from previous structure (EMD-5601) (Taylor et al., 2013) low-pass filtered to 60 Å with SPIDER (Shaikh et al., 2008).

### Statistical analysis

All the biochemical assays were repeated more than three times and student’s *t*-test was used for statistical analysis. Statistical significance was defined by a two tailed *P*-value of 0.05.

## Electronic supplementary material

Below is the link to the electronic supplementary material.

Supplementary material 1 (PDF 698 kb)

## Abbreviations

dsRBD: double-stranded RNA-binding domain
EMSA: electrophoresis mobility shift assay
hDicer: human Dicer
