# Supplementary material for: Structure of precursor microRNA’s terminal loop regulates human Dicer’s dicing activity by switching DExH/D domain
Source: Protein Cell. 2014 Dec 31;6(3):185–93. doi: 10.1007/s13238-014-0124-2 (PMC4348242; doi:10.1007/s13238-014-0124-2)
Supplement: Supplementary file 1 — Supplementary material 1 (PDF 698 kb) [file 13238_2014_124_MOESM1_ESM.pdf]

Supplementary Information for:

Structure of precursor microRNA's terminal loop regulates human Dicer's dicing activity by switching DExH/D domain

Zhongmin Liu[1,2], Jia Wang[2], Gang Li[1]*, Hong-Wei Wang[2]*

[1]Department of Biochemistry and Molecular Biology, School of Basic Medical Sciences, Peking University Health Science Center, Beijing 100191, China;

[2]Tsinghua-Peking Joint Center for Life Sciences, School of Life Sciences, Tsinghua University, Beijing 100084, China

All correspondence should be addressed to: Hong-Wei Wang (hongweiwang@tsinghua.edu.cn) or Gang Li (ligang55@bjmu.edu.cn)

Supplementary Figures

Supplementary Fig 1

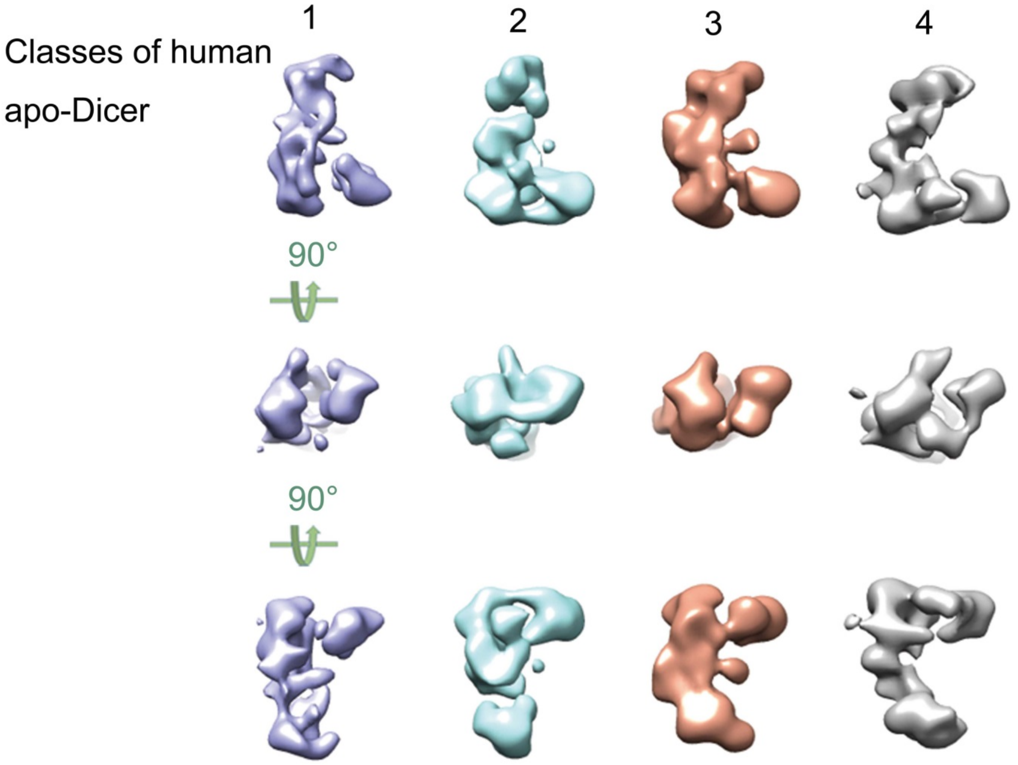

Supplementary Fig 1. 3D Classes of human apo-Dicer. Human apo-Dicer images were classified into four classes from which 3D reconstructions were obtained as shown in columns 1-4, respectively.

Supplementary Fig 2

A

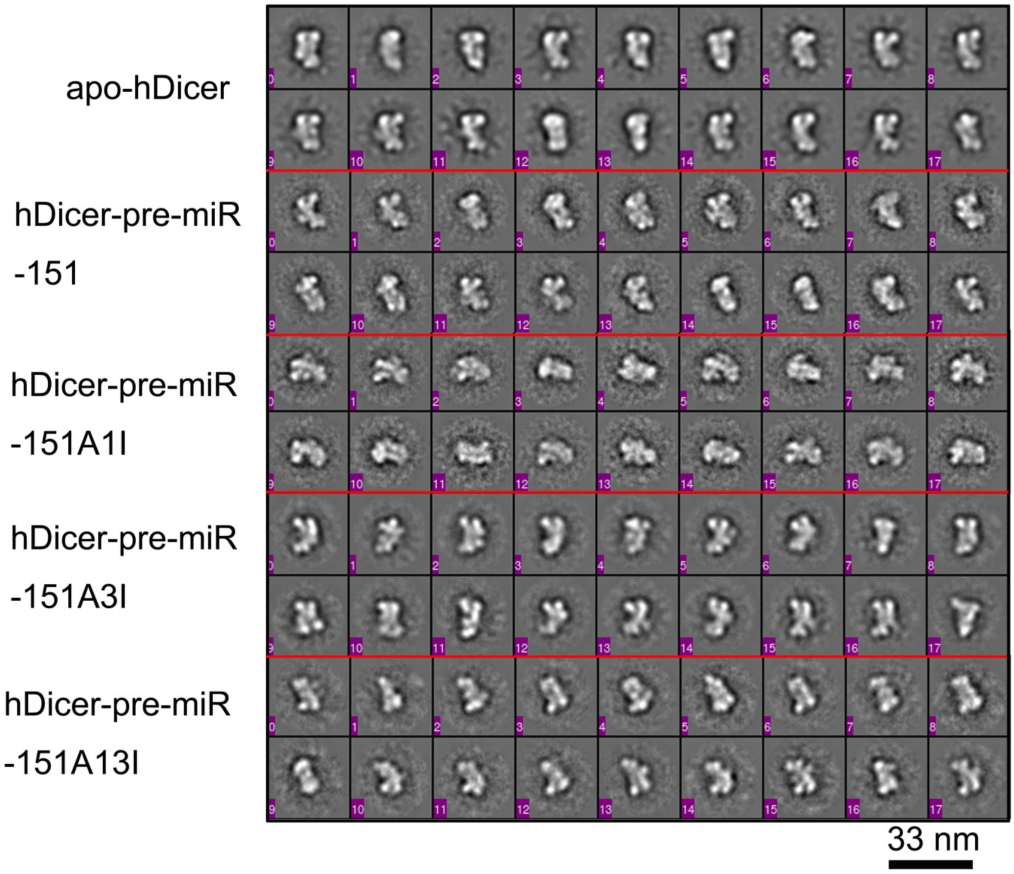

B

Sample	Resolution	Particles percentage (%)
Apo-hDicer	16.8	28.8
hDicer-pre-miR-151	19.5	25.5
hDicer-pre-miR-151A1I	17.0	25.6
hDicer-pre-miR-151A3I	19.0	22.7
hDicer-pre-miR-151A13I	18.5	25.2

Supplementary Fig 2. Classical 2D class-averages and 3D classification information of human Dicer with and without RNA substrates.
